# A New Radiological Parameter in Pediatric Lateral Condyle Fractures: How Effective Is Fragment Size?

**DOI:** 10.3390/medicina62061123

**Published:** 2026-06-09

**Authors:** Tayfun Aman, Batuhan Gencer, Necdet Sağlam, İsmail Türkmen

**Affiliations:** 1 Orthopaedics and Traumatology Clinic, University of Health Sciences, Sancaktepe Şehit Prof. Dr. İlhan Varank Training and Research Hospital, 34785 Istanbul, Turkey; 2 Department of Orthopaedics and Traumatology, Marmara University Pendik Training and Research Hospital, 34899 Istanbul, Turkey; gencer.batuhan@gmail.com; 3 Acıbadem Kartal Hospital, Department of Orthopaedics and Traumatology, 34873 Istanbul, Turkey; necdetsaglam@hotmail.com; 4 Center for Musculoskeletal Surgery Orthopaedics and Traumatology, Klinikum Osnabrück GmbH, 49076 Osnabrück, Germany; dr.ismailturkmen@gmail.com

**Keywords:** lateral condyle fractures, fragment size, lateral bump, humerus lateral condyle

## Abstract

*Background and Objectives*: This study was conducted to investigate the effects of fragment size on clinical and radiological outcomes and the development of complications during the surgical treatment of pediatric lateral condyle fractures. *Materials and Methods*: This retrospective cohort study evaluated data from 47 pediatric patients with lateral condyle fractures, including demographic information, fracture type, range of motion, complications, radiographic assessments, capitellum size, and presence of a lateral bump. Clinical evaluations were performed using the Hardacre functional classification. To objectively investigate the effect of fragment size on prognosis, the total anterior–posterior (AP) and lateral fragment areas, as well as the corresponding AP and lateral capitellum areas (to provide an individualized constant for ratio calculation), were measured. The fractured fragment/capitellum area ratio was then calculated by dividing the fragment area by the respective capitellum area. All statistical analyses were conducted using a two-tailed significance threshold of *p* < 0.05, and corresponding 95% confidence intervals were calculated where applicable. *Results*: After a mean follow-up of 66 months (range: 12–142 months), no significant association was identified between the fractured fragment/capitellum area ratio and either the range of motion (*p* > 0.05) or the presence of a palpable lateral bump (*p* > 0.05). In contrast, a higher fractured fragment/capitellum area ratio was found to be significantly associated with the presence of radiographic complications (*p* = 0.01) and a larger final capitellar area (*p* = 0.02). *Conclusions*: The fractured fragment/capitellum area ratio, a newly defined parameter for quantifying initial fracture size in pediatric lateral condylar fractures, demonstrated no measurable effects on clinical outcomes, the presence of a lateral bump, or range of motion; however, it was significantly associated with the development of radiological complications and may represent an important predictor of subsequent capitellar hypertrophy. *Level of Evidence*: This study corresponds to Level III—retrospective cohort study.

## 1. Introduction

Lateral condyle fractures of the humerus are a common pediatric peri-elbow fracture requiring surgery and are second only to supracondylar humeral fractures in terms of incidence, with an incidence of 12% [1,2,3]. Treatment methods range from simple immobilization in the case of non-displaced fractures to closed reduction and internal fixation (CRIF) in stable fractures and open reduction and internal fixation (ORIF) in unstable fractures with displacement [2,4,5,6]. The most commonly used material for the fixation of fractures in open surgery is Kirschner wires (K-wires), ideally positioned to cross on the lateral side of the metaphysis [1,2,6]. Approximately 4 weeks after the operation, the wires can be removed, and protected active movement can commence within 2 to 3 weeks.

Although some experts recommend ORIF for Milch type II and III fractures, surgical decision-making based on the use of radiological classifications, such as Milch and Jakob, remains controversial [2,7]. Furthermore, lateral condyle fractures may be associated with various complications during long-term follow-up [7,8,9,10,11,12,13,14]. Therefore, orthopedic surgeons are conducting research to identify the factors that influence prognosis, clinical and radiological results, and the development of complications associated with these fractures.

Several studies have investigated the correlation between fracture crack width, fracture area or displacement, degree of fracture healing, and functional outcomes in regard to various fracture types [15,16,17,18]. Gencer et al. [16] reported the prognostic influence of the initial fracture area on distal tibial pilon fractures. Jiang et al. [17] found a significant correlation between fracture displacement and nonunion in subtrochanteric femur fractures. Furthermore, Rakonjac and Brdar discovered that the width of the initial fracture was associated with stability in pediatric supracondylar humeral fractures [18]. The fundamental principle underlying the conclusions of these studies is that increasing fragment size or the degree of fragmentation may hinder fracture reduction, compromise the local blood supply, and increase stress on implants, thereby predisposing the patient to healing-related complications. Although pediatric patients possess important biological advantages in fracture healing, including a high remodeling capacity and a thick periosteum, the possibility that similar biomechanical and biological mechanisms operate in pediatric fractures, and that fragment size may therefore carry prognostic significance in this population, should not be overlooked. However, to the best of our knowledge, no previous studies have investigated fragment size or fracture area and their correlation with clinical and radiological outcomes in pediatric lateral condyle fractures.

As such, we conducted this study to investigate the effects of fragment size on clinical and radiological outcomes and the development of clinical and radiological complications in the surgical treatment of pediatric lateral condyle fractures.

## 2. Materials and Methods

A retrospective evaluation was performed on 69 pediatric patients diagnosed with lateral condyle fractures between 2007 and 2017 and treated surgically at a tertiary care training and research hospital. The study included all patients < 18 years of age who were diagnosed with an isolated lateral condyle fracture, treated surgically, and completed the diagnosis and treatment processes at the study hospital. Patients were excluded if appropriate imaging modalities were not available in the hospital records and/or archive system (*n* = 5), if they had additional fractures (*n* = 5), or if they failed to attend regular and/or last control follow-up (*n* = 12). Based on the inclusion and exclusion criteria, 22 patients were excluded, and data from 47 were included in the study.

Radiological evaluation was performed using anteroposterior (AP) and lateral radiographs. While a displacement greater than 2 mm, which is defined as the widest distance between the displaced fragment of the lateral condyle and the humerus on anteroposterior and lateral radiographs, or the presence of rotational malalignment was considered an indication for surgery, anatomical reduction was defined intraoperatively as the restoration of satisfactory alignment and articular congruity on standard fluoroscopic anteroposterior and lateral views. However, it should be noted that subtle rotational malalignment may not have been fully apparent with this method. All operations were performed by the same surgical team, using CRIF as the preferred initial approach in all patients. Anatomical reduction was assessed using intraoperative stability and fluoroscopic imaging. Arthrography was not performed in any patient. In cases where anatomical reduction could not be achieved with CRIF, where visible displacement persisted, where rotational malalignment was detected under fluoroscopy, or where joint congruity could not be restored by closed means, open reduction was performed using a Kocher incision to achieve anatomical reduction, adequate alignment, and complete restoration of joint integrity. Two Kirschner wires, either 1.6 mm or 1.8 mm thick, were used as fixation materials and set in one of three positions (divergent, convergent, or parallel), as described in previous reports [1,2,6,10,11,12,13]. Two-wire fixation was preferred in all cases, and a third K-wire was used in cases where instability was suspected. All patients were followed up with a long-arm splint postoperatively, regardless of the fixation method used.

Demographic data such as age, gender, side, fracture type based on Milch and Jakob Classifications, reduction type (CRIF or ORIF), and number and orientation of K-wires used during surgery were recorded retrospectively. Subsequently, all patients and their relatives were contacted by telephone using the phone numbers documented in their patient files and invited to attend the outpatient clinic for the final follow-up. During each patient’s outpatient clinic visit, the examiner (T.A.) performed a thorough physical examination. Using a universal goniometer, the examiner measured the flexion, and extension ranges of motion of both elbow joints and recorded the results. Additionally, all patients were checked for the presence of a palpable lateral bump, which was assessed clinically through palpation and recorded if present. After all clinical evaluations, the patients were categorized according to the Hardacre functional classification based on their range of motion, presence of symptoms, and radiographic findings [19,20].

After the comprehensive clinical evaluation, bilateral elbow X-rays were requested for the entire cohort of patients. All radiographs were obtained in accordance with the institution’s standardized imaging protocol, encompassing routine anteroposterior and lateral elbow radiographs acquired in standardized positioning. The occurrence of radiological complications was evaluated through the analysis radiographs. Radiological complications were defined as the presence of postoperative structural abnormalities, including joint-line irregularity (disruption or incongruity of the articular surface contour), fishtail deformity (central distal humeral metaphyseal osseous resorption resulting in a characteristic V-shaped deformity), and cubitus varus (coronal plane angular deformity characterized by varus malalignment of the elbow). The radiological findings were initially analyzed as a composite outcome because all represent recognized postoperative structural complications following pediatric lateral condyle fractures. The limited sample size precluded adequately powered primary analyses for each complication as an independent endpoint. After the complication analysis, the carrying angle and Baumann angle were measured and recorded on both preoperative and postoperative radiographs. All measurements and examinations were performed by the same two authors, who were blinded to the patients’ clinical outcome status to minimize measurement bias. In instances of incomplete capitellar ossification, measurements were derived from the visible ossified radiographic margins of the capitellum, with anatomical boundaries determined according to the most clearly identifiable contour on standard projections, thereby ensuring measurement consistency. In such cases, the determination of boundaries was established through joint assessment and consensus of both evaluators for the purpose of enhancing measurement reliability. The measurements were repeated by the same authors one week later, and intra-observer and inter-observer agreement on the radiological measurements were evaluated. The measurement parameters demonstrated high levels of both intra-observer and inter-observer reliability (r > 0.9 and *p* < 0.001 for each) (Table 1).

**Figure 1:**
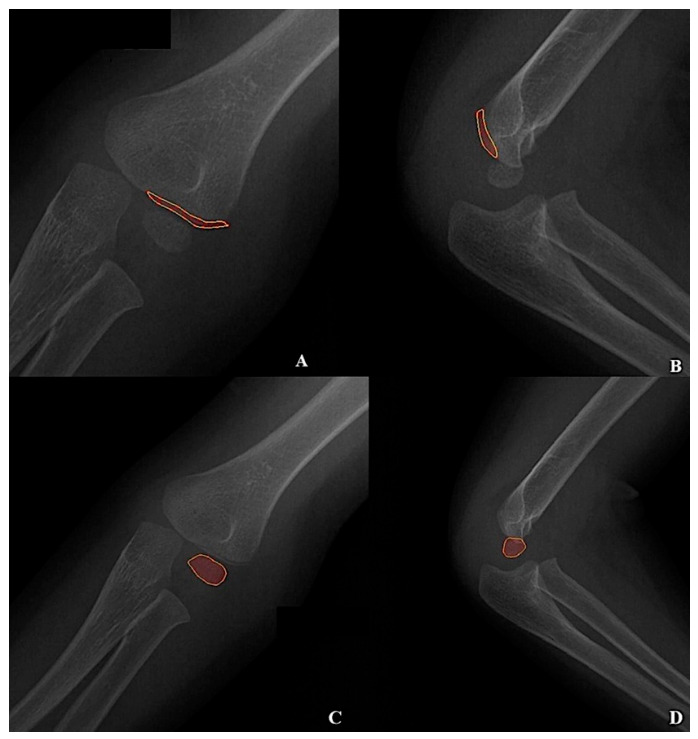
Elbow X-ray. (**A**). Measurement of fracture fragment area on AP radiograph. (**B**). Measurement of fracture fragment area on lateral radiograph. (**C**). Measurement of capitellum area on AP radiograph. (**D**). Measurement of capitellum area on lateral radiograph.

**Figure 2:**
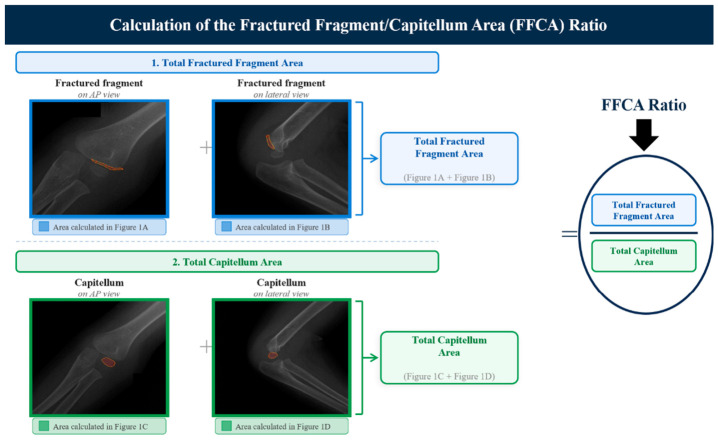
Diagram showing measurement of ratio of fractured fragment/capitellum area.

The ExtremePacs System (Version 4.3, Ankara, Turkey) was used to accurately calculate the radiological measurement of the area of the osseous fragment distal to the fracture line on the AP and lateral radiographs (Figure 1A,B). The total fractured fragment area was calculated by multiplying the areas obtained from the AP and lateral radiographs. Importantly, this parameter is affected by even slight changes in shooting distance, shooting angle, and arm position. To eliminate the effect of these variables and to obtain a more objective parameter, proportional evaluation was preferred. The area of the capitellum in the initial AP and lateral radiographs was measured (Figure 1C,D) and multiplied to calculate the total capitellum area. Only then was the ratio of the fractured fragment/capitellum area (FFCA) calculated by dividing the two values. It was then used to accurately evaluate the size of the fractured fragment (Figure 2). It is imperative to emphasize that the FFCA ratio is to be interpreted as an empirical radiographic index. As explained above, rather than being a direct anatomical measurement, this ratio is calculated by multiplying the coronal and sagittal projections into a dimensionless comparative parameter. While the combination of projected measurements from different radiographic planes does provide a standardized comparative metric, its biological interpretation remains conceptual.

To objectively evaluate changes in capitellar size on direct radiographs, the final capitellar area ratio was calculated by measuring the capitellar area on anteroposterior and lateral radiographs of both elbows at the final follow-up. The measurements were multiplied to obtain the total capitellar area for the fractured and intact sides, and the ratio was calculated by dividing the fractured side value by the intact side value. Calculating the final capitellum area ratio (FCAR) enabled us to evaluate dimensional changes in the capitellum, such as shrinkage due to avascular necrosis or hypertrophy-induced growth.

Statistical analyses were performed using SPSS version 21.0 (IBM Corp., Armonk, NY, USA) for Windows (Microsoft Corp., Redmond, WA, USA). Quantitative variables are presented as medians (minimum–maximum values), as the data distribution was skewed. Because the data were not normally distributed, comparisons between groups were performed using the nonparametric Kruskal–Wallis H test and the Mann–Whitney U test. Post hoc analyses were also conducted using the Mann–Whitney U test. The nonparametric Spearman rank correlation test was used to assess inter-observer and intra-observer reliability, as well as correlations between specific variables. In the reliability analysis, correlation coefficients with r > 0.9 were considered to indicate high reliability. Fisher’s exact test and the chi-square test were used to evaluate differences between proportions and associations between categorical variables. All statistical analyses were conducted using a two-tailed significance threshold of *p* < 0.05, and corresponding 95% confidence intervals were calculated where applicable. In correlation analyses designed to detect relationships with medium (0.3) to large (0.5) effect sizes, a sample size of 45 patients is required to achieve 80% statistical power.

## 3. Results

Of the 47 patients (mean ± SD age, 9.68 ± 3.49 years) treated surgically, “closed” anatomical reduction was achieved in only 8 (17%), whereas 39 (83%) underwent ORIF. In the majority of cases, two-wire fixation was sufficient for stability, with a third wire considered necessary in only six (12.8%) patients. Of the wires used, 34 (72.3%) were divergent, 9 (19.1%) were convergent, and 4 (8.5%) were parallel. Details regarding the demographic profiles of the patients and the characteristics of the operations are provided in Table 2.

With a median follow-up period of 66 months (range: 12–142 months), the median elbow flexion was 135° (range: 130–145°), and the median extension was −5° (range: −10–0°). No radiological complications were observed in 21 patients (44.7%), whereas cubitus varus was identified in 3 patients (6.4%), joint-line irregularity was observed in 20 patients (42.5%), and fishtail deformity was found in 3 patients (6.4%). No patients had a poor outcome according to the Hardacre classification, and 26 patients (55.3%) achieved an excellent outcome. The median ratio of the fractured fragment/capitellum area was calculated to be 0.3 (0.03–3.14). No significant relationship was observed between elbow flexion (*p* = 0.374) and extension (*p* = 0.442) ranges of motion and the FFCA ratio. In contrast, there was a statistically significant difference between the presence of radiological complications and the FFCA ratio, with such complications occurring more frequently in patients with higher FFCA values (*p* = 0.01). Post hoc analyses demonstrated that an increased FFCA ratio was not associated with an increased risk of fishtail deformity (*p* > 0.05). However, higher FFCA ratios were associated with an increased risk of joint-line irregularities and cubitus varus deformity (*p* < 0.05 for each), with the highest ratios observed in patients who developed cubitus varus (median FFCA, 1.19; range, 0.24–3.14). Moreover, a significant positive relationship was found between the FFCA and the FCAR, such that higher FFCA values were associated with higher FCAR values (*p* = 0.02). No significant associations were detected between the FFCA ratio and other clinical or radiological parameters (all *p* > 0.05). Finally, a clinically palpable lateral bump was detected on physical examination in 24 patients (51.1%). No statistically significant association was observed between the presence of a clinically palpable lateral bump and the FFCA ratio (*p* = 0.596). Detailed clinical and radiological outcomes and their relationships with the fractured fragment/capitellum area ratio are provided in Table 3.

## 4. Discussion

Orthopedic surgeons continue to research factors that influence the prognosis of pediatric lateral condyle fractures, including their impact on functional and radiological outcomes. Although several previous studies have investigated the correlation between fracture crack width, fracture area or displacement, and functional outcomes in various fracture types, to the best of our knowledge, no such radiological parameters associated with lateral condyle fractures have been described. In this context, our study’s strength and its contributions to the literature are evident. The most important finding of our study was the identification of a significant association between the fractured fragment/capitellum area ratio, a novel radiological parameter that can be calculated from initial radiographs, and the development of radiological complications, specifically joint-line irregularities and cubitus varus deformity.

In our study, satisfactory ranges of motion of elbow flexion and extension and good-to-excellent functional results, according to the Hardacre functional classification, were obtained with a minimum follow-up of one year and a mean follow-up of five years. Pediatric lateral condyle fractures have been associated with satisfactory functional outcomes in the literature as well [7,10,21,22]. Song et al. [7] reported excellent functional outcomes based on the Hardacre functional classification, and Weiss et al. [21] found that the degree of displacement and fracture classification had no effect on functional outcomes. However, in contrast to these results, in a study by Sinikumpu et al. [9] in 2017, 42 children with a history of lateral condyle fracture developed elbow range-of-motion limitations regardless of the treatment performed, with a mean follow-up of 12.4 years. In our study, we found that joint function was independent of fracture classification and fragment size (*p* > 0.05). We believe that the most important parameter in achieving satisfactory postoperative range of motion is the quality of reduction. We aimed to achieve absolute anatomical reduction, which is defined as the restoration of satisfactory alignment and articular congruity on standard fluoroscopic images; however, we acknowledge that subtle rotational malalignment may not have been fully apparent with this method. In fact, the relatively high conversion rate from closed to open reduction observed in this study (83%) can be attributed to the strict emphasis placed on achieving anatomical reduction. In instances where even minimal uncertainty persisted during fluoroscopic assessment, no additional risk was deemed acceptable, and conversion to open reduction was initiated to ensure optimal alignment. If anatomical reduction is successful, satisfactory functional results can be achieved in pediatric lateral condyle fractures regardless of fragment size. It is important to emphasize that, although our minimum follow-up of one year and mean follow-up of five years were sufficient for evaluation, Sinikumpu et al. [9] reported a limitation of motion after a mean follow-up of 12.4 years. Considering that children’s bony structures are still growing and developing, different results may be observed after long-term follow-up. A more objective assessment may be possible with long-term follow-up to evaluate the quality of reduction, fracture classification, and fracture fragment size.

Several complications can follow surgical treatment of lateral condyle fractures, such as nonunion, avascular necrosis, fishtail deformity, joint-line irregularities, and shrinkage of the capitellum due to avascular necrosis or hypertrophy of the capitellum due to physeal reactivity [7,8,9,10,11,12,13,14]. In our study, we observed radiological irregularity in the joint line in 20 (42.5%) children, fishtail deformity in 3 (6.4%) children, and cubitus varus in 3 (6.4%) children. Similar findings have been reported in other studies. In 2012, Cates et al. [23] investigated capitellum physeal arrest using computed tomography and reported a complication rate of 66%. In 2009, Weiss et al. [21] reported a complication rate of approximately 25% after lateral condyle fractures in a study involving 158 patients. Complications after lateral condyle fractures are associated with impaired blood supply, inadequate remodeling capacity, and physeal avascularity or reactivity after surgery [7,8,9,10,11,12,13,14]. However, to the best of our knowledge, no previous study has investigated the relationship between fragment size and complications. In this regard, the effect of the recent introduction of the technique of calculating the fractured fragment/capitellum area ratio is unique. The findings of this study suggest that the presence of radiological complications is closely related to the ratio of fractured fragment/capitellum area. Higher FFCA ratios appear to be associated with an increased risk of radiological joint-line irregularities and the development of cubitus varus deformity, indicating that this parameter may have prognostic value in early radiological assessment. Moreover, a statistically significant relationship was observed between the ratio of the fractured fragment/capitellum area and the final capitellum area ratio. Patients with higher fractured fragment/capitellum area ratios had higher final capitellum area ratio values (*p* = 0.02). The final capitellum area ratio is a parameter calculated to evaluate changes in the size of the capitellum independently of parameters such as shooting distance and arm position. In our study, we determined the final capitellum area ratio to be 1.38 ± 0.66, meaning that the size of the capitellum tended to increase (hypertrophy) on the fractured side. More importantly, given the significant relationship between them, we believe that a fractured fragment with a larger area is an important factor in hypertrophy of the capitellum that may develop in the future.

When considering why larger fracture fragments may be associated with increased radiological complications without adversely affecting clinical outcomes, the primary factor is the potential difficulty in achieving anatomical reduction due to the larger fragment area. However, because the quality of reduction influences both radiological complications and clinical outcomes, and because we tried to achieve anatomical reduction with all patients in our study using either closed or open techniques, this factor alone is unlikely to fully explain the observed findings. Another important consideration is that increasing the fracture fragment size may result in greater mechanical stress on the fixation construct, particularly the Kirschner wires, which may adversely affect fracture stability and healing. Furthermore, a larger fracture surface area may be associated with a more prolonged inflammatory response and the disruption of local vascularity, leading to impaired fracture biology and delayed radiological healing [16]. Nevertheless, a fundamental question remains: why do these factors not result in inferior clinical outcomes? In pediatric patients, several biological factors play a predominant role in mitigating these effects, including a high remodeling capacity, robust vascularization potential, thick periosteal layer, and relatively strong ligamentous structures [24]. These characteristics facilitate fracture healing, increase tolerance to radiological abnormalities, and attenuate their clinical impact. Nevertheless, despite the absence of immediate clinical consequences, underlying radiological complications may persist and potentially serve as a substrate for future pathological changes, such as hypertrophy of the capitellum. It is also important to emphasize that whilst it may be hypothesized that larger fracture fragments could potentially influence local vascular dynamics, this proposed mechanism remains speculative and was not directly investigated in this study.

One of the most common complaints from patients and their relatives during the long-term follow-up after lateral condyle fracture is the presence of a palpable lateral bump. In our study, palpable lateral bumps were observed in 24 (51.1%) patients. In a study by Weiss et al. [21], a lateral bump was detected in 46% of patients. In a 2017 review by Shaerf et al. [13], the presence of a lateral protrusion (i.e., “bump”) was found to be the most common complication following this type of fracture (73%), and the authors hypothesized that it was due to coronal malrotation of the distal fragment. In 2010, Koh et al. [25] reported a lateral protrusion (i.e., “bump”) in 22.3% of patients and found that such a bump was more common in patients with Jakob Type 2 and 3 fractures. In contrast, no correlation was found between the presence of a bump and any other parameters in our study. When we compared the presence of a lateral bump with the Milch and Jakob classifications and the ratio of the fractured fragment/capitellum area, no statistically significant relationship was found (*p* > 0.05 for each). This finding suggests that the presence of a bump is associated with patient-related factors, possibly remodeling capacity, rather than surgeon- or fracture-related factors. In fact, a study by Sinikumpu et al. [9] in 2017 reported the presence of a lateral bump in 6 of 32 patients with an average follow-up of 12 years and found that this bump was not remodeled during long-term follow-up.

We acknowledge that this study and the newly defined rate do not necessarily constitute the generation of a treatment algorithm or the definition of a new indication for pediatric lateral condyle fractures. A pivotal aspect of the treatment of pediatric fractures is the provision of information to families regarding the prognosis of the fracture and its potential complications. We hypothesized that the FFCA ratio may serve as a potential prognostic indicator. Although the FFCA ratio demonstrated associations with certain radiological outcomes in this cohort, the clinical significance of these findings remains uncertain, particularly in the absence of corresponding associations with functional outcomes. With further refinement and integration into computerized systems, this parameter could be explored in future studies as a tool to assist clinicians in conveying possible outcome scenarios to families at the time of initial presentation.

Our study has several limitations. First, its retrospective design and relatively short follow-up period must be acknowledged. The exclusion of 22 out of 69 patients who were initially deemed eligible may have resulted in the introduction of selection bias. As the retrospective study design meant that comprehensive baseline data for excluded patients was not available, a formal comparison between included and excluded individuals could not be performed. Furthermore, physeal changes in pediatric patients may manifest years later as growth progresses and therefore may not have been fully captured in the present cohort. Another important limitation is that both fracture fragment size and radiological healing are influenced by multiple potential confounding factors, including patient age and comorbidities. Moreover, the relatively limited sample size precluded statistically reliable subgroup analyses based on age categories. It is acknowledged that age-related variations in elbow morphology may potentially influence FFCA measurements. The inability to specifically assess this relationship should therefore be considered a limitation of this study. Another limitation is that oblique radiographs were not included in either radiological measurements or operative decision-making. Although the literature suggests that oblique radiographs may be useful in cases requiring intermediate treatment decisions, they were deliberately excluded from measurement analyses to reduce potential measurement bias, considering the difficulty in standardizing oblique radiographic views. An additional limitation that warrants emphasis relates to the method used to calculate the fracture area, which is the main contribution of this study. As mentioned before, the FFCA ratio is to be interpreted as an empirical radiographic index. From a strictly geometric perspective, this approach may be considered insufficient. However, the objective of the present study was not to establish a precise volumetric assessment or to introduce a new dimensional measurement but rather to identify a practical prognostic parameter that can be readily implemented in routine clinical practice. The rationale for evaluating these measurements collectively, rather than independently, is that the areas derived from AP and lateral radiographs should not be regarded as independent variables. Finally, evaluating joint-line irregularities solely using plain radiography and in a binary manner (presence or absence) inevitably introduces a degree of subjectivity into the assessment of radiological complications. Although three-dimensional evaluation of bone and cartilage would be ideal for detecting subtle irregularities, advanced imaging modalities such as computed tomography and magnetic resonance imaging were not utilized because of concerns regarding additional radiation exposure in children and increased healthcare costs.

It is also imperative to acknowledge the inherent statistical limitations of the present study. First, the absence of a formal correction for multiple comparisons may have increased the risk of Type I errors. Second, subgroup analyses were conducted with a very small number of patients, which limits the statistical strength and interpretability of these findings. Consequently, it is imperative to acknowledge that the findings of this study should be regarded as exploratory in nature rather than confirmatory. Furthermore, while Spearman’s correlation is deemed adequate for reliability analysis, the absence of the intraclass correlation coefficient in the reliability analysis of continuous measurements is regarded as a methodological limitation. Finally, the analyses were primarily based on univariate comparisons without adjusted multivariable modeling; therefore, the independent prognostic value of FFCA could not be definitively established, and the potential influence of confounding clinical and surgical factors should be considered when interpreting the findings. Future studies incorporating longer follow-up periods, larger cohorts, and multivariable analyses may allow for a more objective and comprehensive evaluation of these factors.

## 5. Conclusions

The ratio of the fractured fragment-to-capitellum area, a novel parameter introduced to estimate initial fragment size in pediatric lateral condyle fractures, showed an association with the development of certain radiographic complications, particularly joint-line irregularities and cubitus varus, within the present cohort. While this observation may suggest a possible relationship between a larger initial fragment size and less favorable radiographic remodeling, these findings should be interpreted cautiously given the exploratory nature of the analysis. In contrast, achieving anatomical reduction, regardless of fragment size, was associated with favorable functional outcomes. It should be emphasized that the presence of a lateral bump represents a complication independent of fragment size. Further prospective studies with larger cohorts are required to validate these findings and to clarify the role of this ratio in defining risk stratification and its potential utility in patient counseling.

## Figures and Tables

**Table 1:** Analysis of intra-observer and inter-observer reliability.

	Intra-Observer Reliability		Inter-Observer Reliability	
	r *	*p* *	r *	*p* *
Baumann Angle—Preoperative	0.92	<0.001	0.962	<0.001
Baumann Angle—Last Follow-up	0.941	<0.001	0.966	<0.001
Carrying Angle—Preoperative	0.917	<0.001	0.964	<0.001
Carrying Angle—Last Follow-up	0.902	<0.001	0.96	<0.001
Total Fracture Fragment Area	0.905	<0.001	0.962	<0.001
Total Capitellum Fragment Area	0.903	<0.001	0.961	<0.001
Final Capitellum Area Ratio	0.92	<0.001	0.961	<0.001
Ratio of Fractured Fragment/Capitellum Area	0.932	<0.001	0.97	<0.001

* Spearman’s rank correlation coefficient and probability value.

**Table 2:** Demographic profile and operative characteristics of patients.

Demographic Profile and Operative Characteristics		Number of Patients (%) (N = 47)
Age * (years)		9.68 ± 3.49 (4–18)
Gender	Female	14 (29.8%)
	Male	33 (70.2%)
Side	Right	15 (31.9%)
	Left	32 (68.1%)
Milch Classification	Type 1	33 (70.2%)
	Type 2	14 (29.8%)
Jakob Classification	Type 1	15 (31.9%)
	Type 2	23 (48.9%)
	Type 3	9 (19.1%)
Reduction Type	ORIF	39 (83%)
	CRIF	8 (17%)
Number of k-wires	Two	41 (87.2%)
	Three	6 (12.8%)
Orientation of k-wires	Divergence	34 (72.3%)
	Convergence	9 (19.1%)
	Parallel	4 (8.5%)

* Mean ± standard deviation (minimum maximum values).

**Table 3:** Clinical and radiological results and their relationship with the fractured fragment/capitellum area ratio.

Clinical and Radiological Results		Number of Patients	Relationship with the FFCA
Follow-up (months)		66 (12–142)	N/A
Flexion Range of Motion		135° (130–145)	* *p* = 0.374
Extension Range of Motion		−5° (−10–0)	* *p* = 0.442
Hardacre Functional Classification	Excellent	26 (55.3%)	** *p* = 0.41
	Good	21 (44.7%)	
	Poor	0	
Complications	None	21 (44.7%)	*** *p * = 0.01
	Cubitus Varus	3 (6.4%)	
	Irregularity	20 (42.5%)	
	Fishtail	3 (6.4%)	
Lateral Bump	None	23 (48.9%)	* *p* = 0.596
	Yes	24 (51.1%)	
Final Capitellum Area Ratio (FCAR)		1.25 (0.5–3.49)	* *p* = 0.02
Baumann Angle, Last Follow-up		68° (54–91)	* *p* = 0.690
Carrying Angle, Last Follow-up		7° (2–21)	* *p* = 0.489
Total Fracture Fragment Area		980 (120–22575)	N/A
FFCA		0.3 (0.03–3.14)	N/A

* *p*: Statistical significance “*p*” value was calculated through Spearman's Correlation Test. ** *p*: Statistical significance “*p*” value was calculated through Mann-Whitney U Test. *** *p*: Statistical significance “*p*” value was calculated through The Kruskal-Wallis H Test.

## Data Availability

The data presented in this study are available on request from the corresponding author. The data are not publicly available due to the local "Personal Data Protection Law".
